# Inhaler use technique course: an effective postgraduate training solution for pharmacists to enhance therapeutic outcomes as part of patient education

**DOI:** 10.1186/s12909-024-05129-3

**Published:** 2024-02-19

**Authors:** Weronika Guzenda, Jerzy Żabiński, Beata Plewka, Michał Byliniak, Piotr Przymuszała, Piotr Dąbrowiecki, Michał Michalak, Magdalena Waszyk-Nowaczyk

**Affiliations:** 1 Pharmacy Practice and Pharmaceutical Care Division, Chair and Department of Pharmaceutical Technology , Poznan University of Medical Sciences, 3 Rokietnicka Street, 60-806 Poznan, Poland; 2 Department of Organic and Physical Chemistry, Medical University of Warsaw, 1 Banacha Street, 02-097 Warsaw, Poland; 3 Mazovian Pharmaceutical Chamber, 01-882, Żeromskiego 77/6 Street, Warsaw, Poland; 4 Infarma, 182 Pulawska Street, 02-670 Warsaw, Poland; 5 Department of Medical Education, Poznan University of Medical Sciences, 7 Rokietnicka Street, 60- 806 Poznan, Poland; 6 Department of Infectious Diseases and Allergology, Military Institute of Medicine, 128 Szaserów Street, 04-141 Warsaw, Poland; 7 Chair and Department of Computer Science and Statistics, Poznan University of Medical Sciences, 7 Rokietnicka Street, 60-806 Poznan, Poland

**Keywords:** Pharmaceutical care, Asthma, COPD, Pharmacist, Inhalers’ use training

## Abstract

**Background:**

Patients with asthma and chronic obstructive pulmonary disease could benefit from education on using inhalers provided by pharmacists. However, pharmacists may have limited competencies, indicating the necessity to implement appropriate postgraduate courses. The study aimed to evaluate an inhaler use course for pharmacists, including its impact on participants’ knowledge and satisfaction.

**Methods:**

The study involved 261 pharmacists from community pharmacies and was conducted between September 2019 and March 2021. A pre-post analysis of their knowledge of the topic was applied. Additionally, at the beginning of the course, participants were asked about their educational needs, and at the end, they completed a satisfaction survey. The preferred learning formats indicated by participants were interactive workshops and lectures.

**Results:**

As a result of the course, both their actual and self-assessed level of knowledge significantly increased. The percentage of correct answers in the test before the training was 24.4%, while after, it was 84.3% (*p* < 0.0001). Before the course, their average self-assessed level of knowledge was 52.0%, and after the training, it increased to 90.0% (*p* < 0.0001). Almost all respondents stated that the course met their expectations. They estimated their satisfaction at 94.0% and the usefulness of the provided information at 98.0%.

**Conclusions:**

Improved preparation of pharmacists resulting from their participation in the course can contribute to providing more professional advice to patients, thereby positively influencing the pharmaceutical care process in community pharmacies.

## Introduction

Asthma is a prevalent condition that affects individuals across all age groups. In many cases, it leads to a decline in the quality of life due to respiratory issues such as coughing, shortness of breath, or wheezing. The primary treatment for asthma and various respiratory diseases is the use of inhalers [1–3]. However, the correct inhalation technique is crucial for the efficacy of inhalation treatment [4, 5]. There is uncertainty regarding whether patients receive adequate information or proper instructions on how to use inhalers. Previous studies have highlighted a significant number of errors made by patients in their inhalation techniques [6–8]. For instance, a study conducted in Portugal in 2015 assessed patients with asthma or COPD (chronic obstructive pulmonary disease). The results revealed that only 13.4% of the participants demonstrated the correct inhaler usage without making any mistakes. On the contrary, 68.0% of patients using dry powder inhalers (DPI) failed to hold their breath after inhalation, and 61.3% did not exhale deeply before administering the inhaler. Similar errors were prevalent among patients using pressurized metered dose inhalers (pMDI), with 62.5% and 50.0% of the respondents making the same mistakes, respectively. Additionally, nearly 40% of people using pMDI forgot to shake it before starting inhalation [9].

Pharmacists can prevent drug-related problems and help to achieve optimal therapeutic outcomes, particularly in chronic conditions. Increasing evidence suggests that their interventions can reduce patient errors and enhance disease control [10]. In the case of COPD and asthma, patients may lack knowledge or feel uncertain, leading them to seek assistance from pharmacists, as community pharmacies often serve as the initial point of contact between healthcare professionals and patients [11]. Pharmacists indeed hold an invaluable role in this context [12]. Evaluating inhalation techniques, providing education, and, when necessary, referring patients to physicians can be instrumental in helping patients. Hence, establishing proactive and ongoing communication between patients with asthma and COPD with pharmacists, physicians, nurses, and other specialists is essential to implement the best healthcare practices and achieve optimal clinical outcomes. The accessibility of pharmacists also offers a unique opportunity to enhance patient compliance through proper education, monitoring of inhalation techniques, and answering questions affecting treatment results [7, 13]. Consequently, pharmacists’ involvement may play a key role in improving the adherence of patients with COPD and asthma [14, 15]. Furthermore, even if the patients can find instructions for using inhalers online or in drug leaflets, the pharmacist’s important role is to support them in interpreting, understanding, and remembering the obtained information [3, 16, 17].

Unfortunately, studies indicate that the competencies of many pharmacists in providing professional advice and instructions regarding inhaler use may still be limited, impacting their ability to educate patients effectively [17]. Consequently, pharmacists, especially those in community pharmacies, appear to require proper training (additional opportunities to supplement knowledge, such as courses or specializations) to assist patients in reducing errors when using inhaler devices. The primary outcome of pharmacist training is an enhanced standard of patient care. This endeavor produces visible improvements in the quality of patient care [18]. Such educational initiatives would also equip pharmacists with current information on asthma/COPD and appropriate therapies. Additionally, it can enhance their collaboration with physicians by fostering a deeper understanding of specialists’ perspectives and recommendations. Meanwhile, in Poland, as in many countries, pharmacists are mandated to engage in continuous (lifelong) learning according to the Act on the Profession of the Pharmacist from 2020 [19]. The legislation also emphasizes that a pharmacist may provide pharmaceutical care, defined as “(…) a documented process in which a pharmacist, working with the patient and the physician treating the patient, (…), supervises the proper course of individual pharmacotherapy” [19]. However, the education standards for the pharmacist profession do not encompass all practical aspects of pharmacists’ work in a community pharmacy [20, 21]. Additionally, given the novel expansion of pharmacists’ responsibilities in Poland, some may lack updated knowledge due to perceived low competency levels required for new duties [22]. Reports from other countries highlight that too few patients receive comprehensive advice on inhaler usage from pharmacists, possibly stemming from pharmacy employees’ uncertainty about their knowledge. This underscores the importance of workshops as a means to consolidate specific information [23]. In turn, proper training for pharmacists could enhance their ability to effectively educate patients on inhalation techniques [24]. Such consultation should not be limited to verbal communication but should also include a demonstration of the correct technique using a placebo device. Pharmacists should ask patients to replicate the sample inhalation to ensure a correct understanding [25, 26]. Therefore, to better prepare Polish pharmacists for potential future tasks, a continuous learning course on inhaler use was designed and implemented.

The aim of the study was to evaluate the impact of this course on participants’ knowledge and their satisfaction from participating in it. Additionally, participants defined their further educational needs for courses in the use of inhalers.

## Materials and methods

**Fig. 1:**
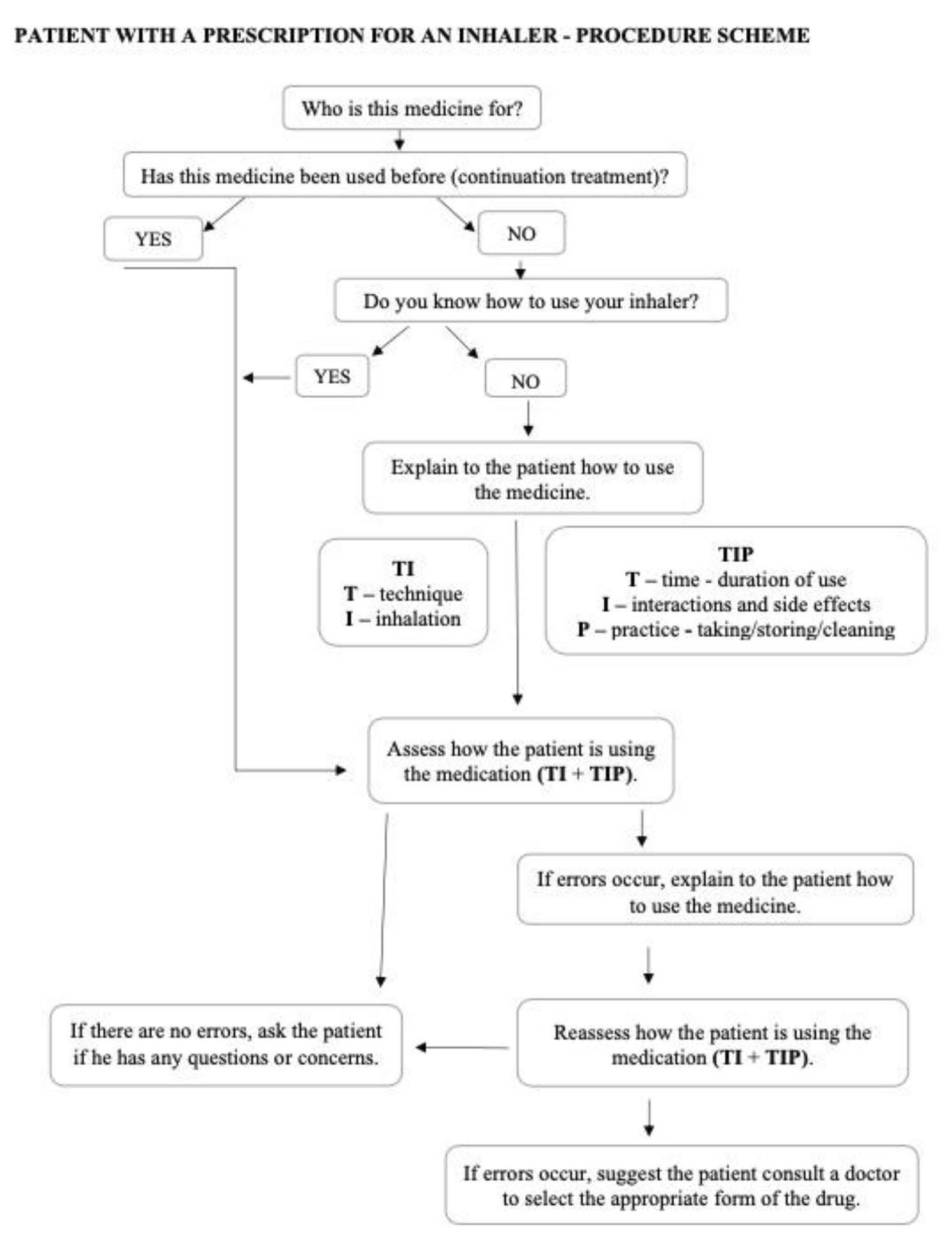
Patient with a prescription for an inhaler - procedure scheme [27]

The study analyzed a course designed to enhance pharmacists’ ability to support patients in using inhalers in community pharmacy, conducted between September 2019 and March 2021 in Warsaw, involving a total of 261 pharmacists. Throughout the course, participants were presented with an original scheme created by the authors of the study for managing patients taking inhaled drugs and providing patient education in the field of inhalation techniques [Fig. 1,^27^]. The presented procedure proposal aims to equip pharmacists working in community pharmacies with tools readily available, enabling them to systematize their interactions with patients using inhalers. In this procedure scheme, when a patient presents a prescription for an inhaler at the pharmacy, it should be ascertained WHO the medication is prescribed FOR. Additionally, it is crucial to assess the patient’s EXPERIENCE with using the inhaler, determining whether the patient has a prior usage history or if this is the first prescription for an inhaler from a physician. In the former scenario, it is advisable to inquire about the patient’s familiarity with the inhaler and demonstrate its usage using a sample version of the medication. In the latter case, if the patient lacks knowledge on how to utilize the medication, a step-by-step explanation of its proper usage should be provided, considering factors such as inhalation technique, duration of use, potential interactions, or side effects [27]. It is noteworthy that this was the first practical training on using inhalers organized in Poland and was carried out in collaboration with the Poznan University of Medical Sciences and the Mazovian Pharmacy Chamber in Warsaw.

In total, six editions of the course were conducted. Each course edition lasted 8 h, divided into a theoretical segment of 4 h for lectures and a practical segment of 4 h for workshops. There was one specific schedule established. First, there were lectures for all participants. Then there was the workshop part: the group was divided in half and worked under the supervision of a specialist. Each edition accommodated approximately 40 participants, with the workshop group of pharmacists divided into two halves, each comprising around 20 participants. The lecture topics covered the etiology and diagnosis of asthma and COPD concerning assisting patients in a pharmacy, practical aspects of pharmacological methods for treating asthma and COPD, principles of selecting inhalers, therapy monitoring, and procedures for providing professional pharmaceutical advice on inhaler use in accordance with the latest asthma and COPD recommendations [28]. During the workshops, participants received training in the use of each type of inhaler and were educated on effectively transferring knowledge to patients. Workshop topics focused on practical aspects of using inhalers (using a demo version), professional pharmaceutical advice on their use, and standard operating procedures (SOP) [27]. Practical education aimed at the proper use of various inhalers, utilizing demonstration models. Initially, the instructor meticulously organized each type of inhaler, providing a step-by-step demonstration of its usage. Subsequently, participants had the opportunity to try to use the inhalers on themselves and pose questions. The entire process was conducted under the instructor’s supervision to guarantee that participants accurately executed the trial inhalations. This model adheres to the principle that pharmacists should first receive a thorough education, enabling them to subsequently educate patients accurately [29]. Participants also received a guide on how to use individual types of inhalers. The practical guide includes a comprehensive description of all types of inhalers, along with step-by-step instructions for usage, accompanied by informational images and the trade names of the medications. Lectures and workshops were conducted by a doctor, a specialist in internal medicine and allergology, and pharmacists specializing in professional advice and pharmaceutical care. The idea for the workshops and lectures resulted from the observations of practice in a community pharmacy, previous conversations with other pharmacists and from Polish Supreme Chamber of Pharmacy, as well as a review of foreign publications and training topics. Additionally, as it was the first such project, in order to broaden the understanding of pharmacists’ further educational needs, surveys regarding the demand for subsequent courses were collected at the beginning. The inclusion criteria for the study were participation in the course, employment in a community pharmacy as a pharmacist, and consent to participate in the study.

Before and after the course, a test was administered to assess participants’ knowledge of the inhaler use technique to evaluate its effectiveness. Additionally, before commencing the training, pharmacists were queried about their expectations regarding an inhaler training course. Furthermore, after completing the course, the trainees filled out a satisfaction questionnaire to assess their experience with the training. The satisfaction survey encompassed various aspects, including the participant’s preference for class type, duration, instructor, coverage of topics, and the type of materials provided.

Obtained data were analyzed using Microsoft Excel, STATISTICA 12 (StatSoft), and STATA14 StataCorp LLC software programs. The analysis process involved the evaluation of pre-post differences in response to test questions and the verification of differences among the respondents in terms of their age and gender. Analyses were conducted, as applicable, with the use of the following tests: the chi-square test of independence and Fisher’s exact test for observed small or zero values, the Wilcoxon signed-rank test, the Mann–Whitney U test, and the Kruskal–Wallis test with Dunn’s post hoc tests. All tests were analyzed at the significance level of a = 0.05 and were considered statistically significant at *p* < 0.05.

The study was approved by the Bioethics Committee No. 602/19 at the Poznan University of Medical Science and was conducted in accordance with the Declaration of Helsinki. Participation in this study was anonymous. All collected data were securely stored in the Department of Pharmaceutical Technology, Pharmacy Practice Division at Poznan University of Medical Sciences.

## Results

**Fig. 2:**
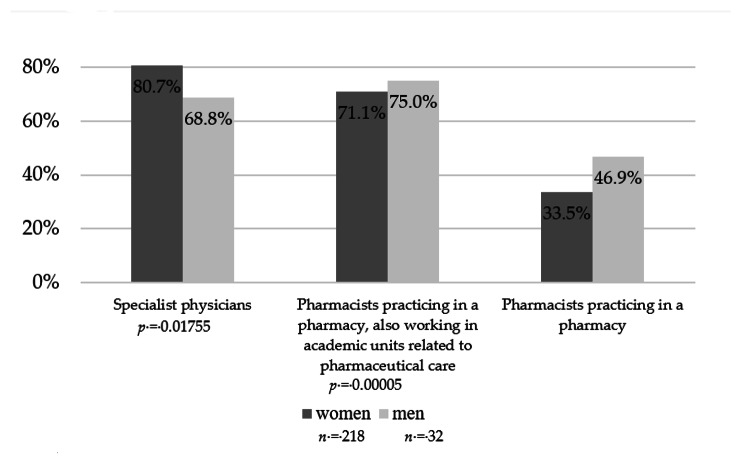
Answers to the question of who should conduct the training by gender (*n* = 250, multiple choice question)

**Fig. 3:**
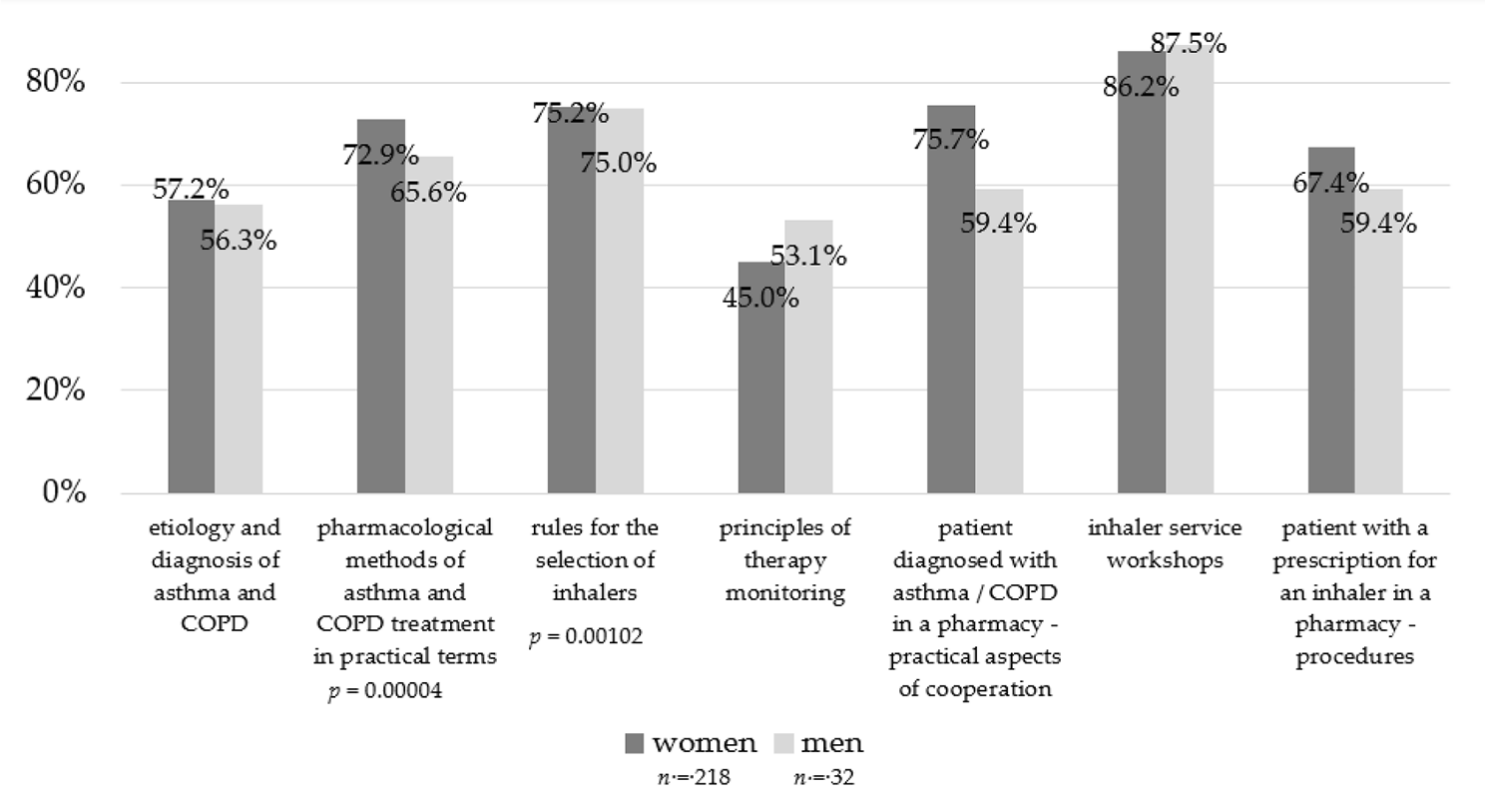
Pharmacists’ interest in the different topics of training, depending on their gender (multiple choice question)

**Fig. 4:**
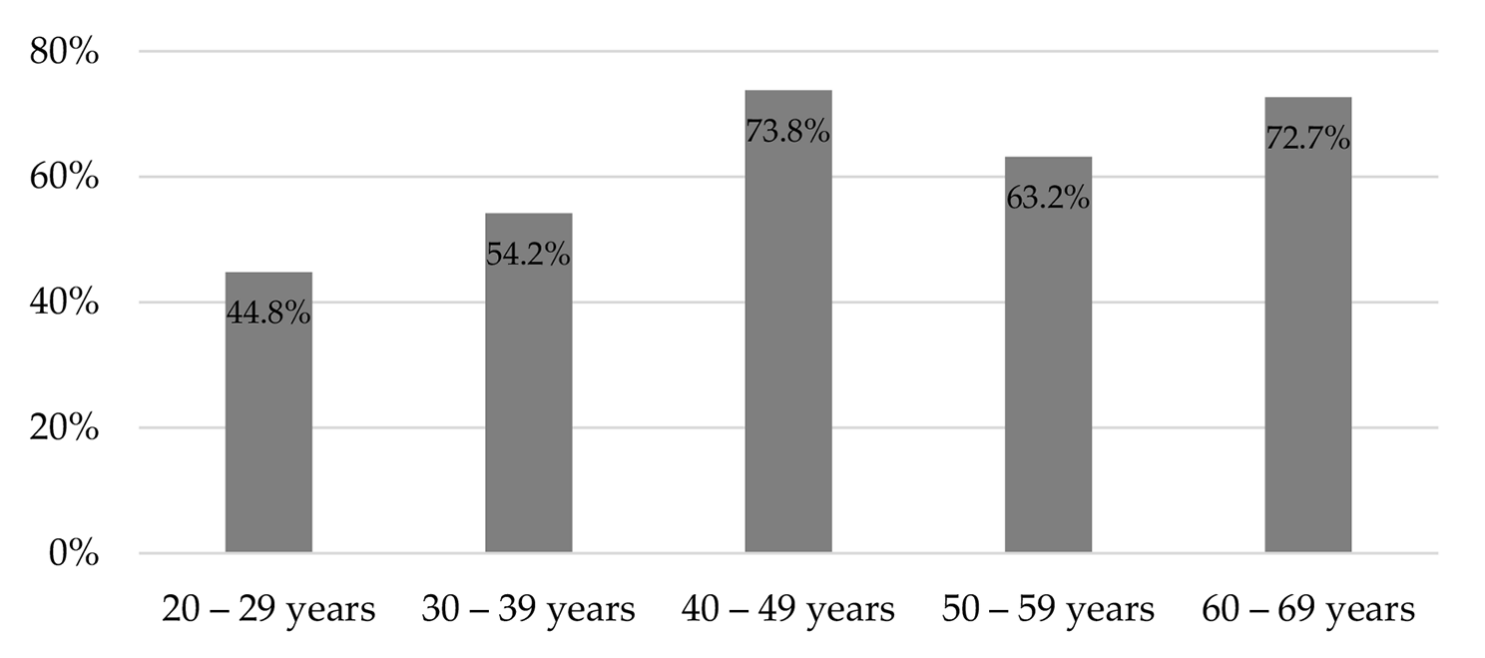
Pharmacists’ interest in the topic of etiology, diagnosis of asthma and COPD, and the possibility of helping patients in a pharmacy depending on their age (*p* = 0.02477)

A total of 260 participants were included in the study (86.9% women and 13.1% men, one person returned an empty questionnaire). Detailed characteristics of pharmacists in terms of age, work experience, specialization, and type of pharmacy in which they work are presented in Table 1. The majority of respondents were in the age group of 30–39 (33.5%), had a specialization (24.9%), and worked in a pharmacy chain of more than six pharmacies (46.2%). In a questionnaire distributed at the beginning of the course to assess their educational needs in the use of inhalers, 81.2% of the respondents stated that they were most interested in interactive workshops and 52.4% in lectures. In comparison, they were less interested in simulated patient methods (42.8%). The trainees were divided regarding the preferred duration of the training: about 4 h − 45.5%, and about 8 h − 41.0%. The respondents also believed that it would be best if the courses were conducted by physicians (79.2%) or pharmacists - academic teachers who also have a practice in a pharmacy (71.6%). However, we noticed some differences in this aspect in terms of respondents’ gender. While women significantly more often than men opted for physicians as instructors (*p* = 0.01755), men more frequently than women chose academic teachers who are also pharmacists (*p* = 0.00005, Fig. 2). Regarding the future subject of the training, the most frequently chosen answers were workshops on the use of inhalers (86.4%), appropriate selection of inhalers (75.2%), and practical aspects of cooperation with a patient diagnosed with asthma / COPD in a pharmacy (73.6%). Some differences were observed here in terms of gender as well. Women exhibited greater interest in the practical pharmacological treatment of asthma and COPD compared to men (*p* = 0.00004) as well as in the principles of inhaler selection (*p* = 0.00102). Detailed division by gender is presented in Fig. 3. Additionally, some age differences were also visible, with participants aged 40–49 and 60–69 expressing greater interest in the etiology, diagnosis of asthma and COPD, and the potential to assist patients in the community pharmacy than other age groups (*p* = 0.024770, Fig. 4).

**Table 1:** Characteristics of participants

Characteristics of participants n (%)			
	Male	Female	Total
Total number of respondents	34 (13.1)	226 (86.9)	260 (100.0)
*Age [years]*	34 (13.5)	217 (86.5)	251 (100.0)
25–29	16 (47.0)	57 (26.3)	73 (29.1)
30–39	11 (32.4)	73 (33.6)	84 (33.5)
40–49	2 (5.9)	41 (18.9)	43 (17.1)
50–59	5 (14.7)	35 (16.1)	40 (15.9)
> 60	0 (0.0)	11 (5.1)	11 (4.4)
*Seniority [years]*	34 (13.2)	223 (86.8)	257 (100.0)
< 5	14 (41.2)	64 (28.7)	78 (30.3)
5–10	12 (35.3)	52 (23.3)	64 (24.9)
11–20	5 (14.7)	54 (24.2)	59 (23.0)
> 20	3 (8.8)	53 (23.8)	56 (21.8)
*Pharmacy specialization*	34 (13.2)	223 (86.8)	257 (100.0)
Yes	6 (17.6)	58 (26.0)	64 (24.9)
No	28 (82.4)	165 (74.0)	193 (75.1)
*Type of pharmacy*	33 (13.3)	216 (86.7)	249 (100.0)
Individual	8 (24.2)	77 (35.7)	85 (34.1)
Chain of up to 6 pharmacies	4 (12.1)	35 (16.2)	39 (15.7)
Chain of more than 6 pharmacies	21 (63.7)	94 (43.5)	115 (46.2)
Another type of community pharmacy	0 (0.0)	10 (4.6)	10 (4.0)

The knowledge test carried out at the beginning and the end showed that the study participants significantly increased their knowledge after participation in the course. The percentage of correct answers in the test was 24.4% before the training and increased to 84.3% following the course (*p* < 0.0001, *n* = 253). The answers to each of the questions are presented in Table 2. In the first question, pharmacists were asked about the interval between each inhalation. Before the training, 33.8% gave the correct answer, and 98.2% after the training. In the second question, pharmacists were asked to name two online databases used to detect potential and real drug problems. Before the training, only 1 person (0.4%) gave the correct answer, and 13.5% provided an incomplete answer. After the training, 90.2% of the respondents gave the correct and full answer. In the third question, pharmacists were asked to list the most common mistakes made by patients when using the pMDI inhaler. The answer was assumed correct when the respondent provided three proper variants. Before the training, 3.8% of respondents were able to accomplish that, and after − 59.1%. In question 4, the respondent was to mention the two most likely side effects that may occur when administering inhaled steroids. Before the training, 4.6% of the respondents gave the correct answer, and after the training, more than half (52.4%). The last two questions were closed. In question 5, the respondent was asked about important factors that patients using DPI inhalers need to be informed about. After the training, more people (81.8%) gave the correct answer compared to the situation before the training (26.5%). Similarly, in the last question about which aerosols are multi-dose inhalers, correct answers were provided by 24.6% of respondents before and 79.6% after the course.

**Table 2:** Pharmacists’ answers to test questions before and after the course

PHARMACEUTICAL TEST - ANSWERS BEFORE AND AFTER (average percentage of correct answers)														
	Question*	1		2		3		4		5		6		
		before	after	before	after	before	after	before	after	before	after	before	after	Total difference
	Total	0.34	0.98	0.07	0.94	0.19	0.82	0.34	0.73	0.27	0.82	0.25	0.80	0.60
		*p* < 0.0001		*p* < 0.0001				*p* < 0.0001		*p* < 0.0001		*p* < 0.0001		
*Gender*	Male	0.44	0.96	0.09	0.86	0.18	0.66	0.26	0.63	0.32	0.82	0.32	0.82	
		*p* < 0.0001		*p* < 0.0001				*p* = 0.0001		*p* = 0.0001		*p* = 0.0001		
	Female	0.32	0.98	0.07	0.95	0.19	0.84	0.35	0.75	0.26	0.82	0.24	0.79	0.61
		*p* < 0.0001		*p* < 0.0001				*p* < 0.0001		*p* < 0.0001		*p* < 0.0001		

*Questions:

1. If the doctor has prescribed two inhalations of the drug at a time, then there should be a break of about…… before administering the next dose

2. List the names of two online databases used to detect potential and real drug problems

3. List three of the most common mistakes patients make when using the pMDI inhaler

4. When using a face mask with an inhalation chamber to administer inhaled steroids, be aware of the risk of side effects. List two most likely side effects

5. What should be recommended to a patient using a DPI inhaler during education?

6. Which of the following aerosols are multi-dose inhalers?

**Fig. 5:**
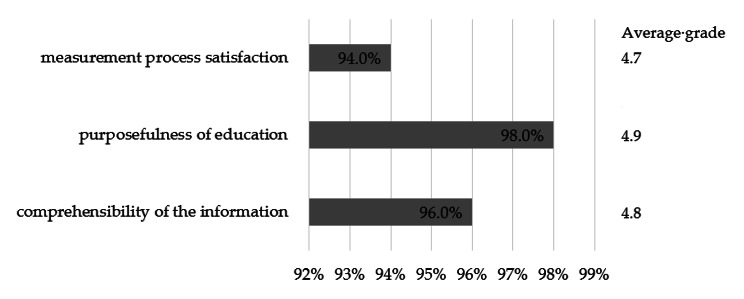
Summary of the respondents’ satisfaction level concerning the implemented training (*n* = 215)

**Fig. 6:**
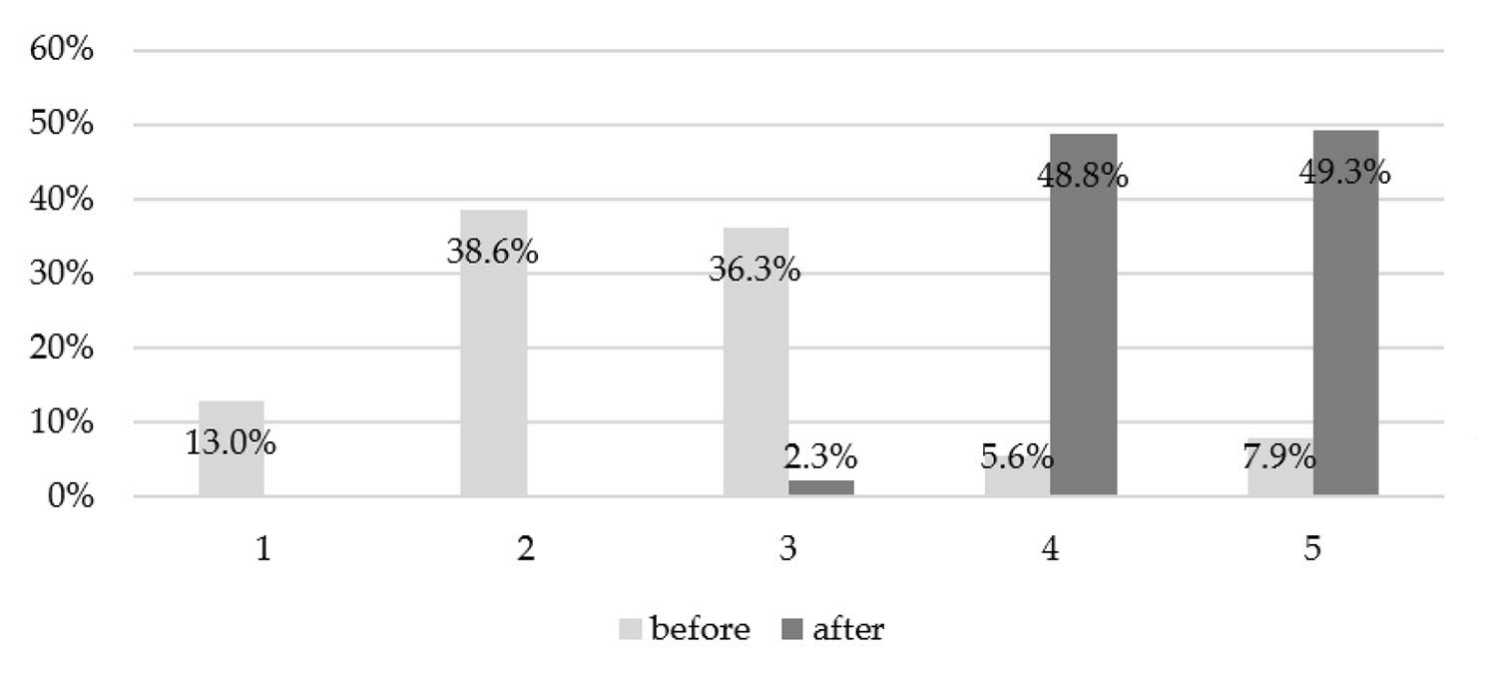
Summary of the pharmacists’ knowledge self-assessment before and after the training (*p* < 0.0001)

At the end of the training, the willing respondents filled out a satisfaction questionnaire, assessing the intervention and the knowledge acquired due to it. 98.6% of people considered that the conducted training met their expectations. The respondents estimated their satisfaction with the course at 94.0% (4.7/5) and the usefulness of the provided information at 98.0% (4.9/5). The intelligibility of the transmitted messages was 96.0% (4.8/5). A summary of the average of all assessments given by the respondents is presented in Fig. 5. Pharmacists could also assess the state of their knowledge before and at the end of the education. Before the training, the average level of self-assessed knowledge was 52.0%, and after the training, it was 90.0% (*p* < 0.0001). Detailed results are presented in Fig. 6.

## Discussion

The literature underscores the importance of consistency in applied educational solutions, particularly in the context of pharmacists’ continuous education [29–32]. In Poland, aligning with evolving laws and new responsibilities for pharmacists, there is growing pressure for additional education, encompassing courses, training, postgraduate studies, or specializations [33]. Hence, to support these and future similar interventions, our study also assessed the demand for it. During the courses, a pivotal aspect was the opportunity for collaboration between pharmacists and physicians, involving the exchange of experiences, complementing each other’s roles, and discussing the same matter from two different perspectives. As anticipated during the planning of the course presented, interactive workshops and lectures emerged as the most commonly preferred formats for continuous learning on the subject. In our study, we organized a format in which pharmacists could acquire not only theoretical knowledge about asthma and COPD but also practical information on the use of inhalers, with the opportunity to practice their skills using demonstration versions. This was crucial as participants are likely to be more engaged in the learning process when it is problem-centered, practical, and relevant to their professional lives [34]. Interestingly, fewer than half of the respondents selected simulation patients as their preferred learning format. This could be attributed to a lack of prior experience working with this method or a fear of the unknown. Admittedly, simulation methods were in the initial stage of adoption in Poland, especially in the education of pharmacists [35]. During the research period, the first professionally equipped, modern Medical Simulation Center in Poland was being expanded in Poznań [36]. It’s also possible that pharmacists, aware of their limited competencies on the subject, preferred a ‘safer’ format instead of simulation where they might have to interact with a simulated patient, for instance. Hence, the utilization of simulation methods could be considered in later stages of such courses once participants have already acquired some knowledge and skills on the subject. During the workshops, the respondents were also asked about their expectations, including the duration of the training or future postgraduate education topics. Collecting data from respondents regarding their expectations allows adjusting the course to participants’ expectations to enhance efficiency, enabling participants to extract as much useful information as possible [37]. When asked about future training topics, most participants chose inhaler technique workshops, confirming that the course designed by the research team seemed to meet their expectations. This illustrates a significant demand for this type of practical training and indicates that the research team correctly identified a niche in the proposition of continuous education courses for pharmacists.

As evidenced by the knowledge test administered before and after the course, pharmacists’ skills significantly improved following their participation in training. Their initially low level of knowledge on the topic can be attributed to various factors, including the potential forgetting of education acquired during studies due to a lack of refreshing, the teaching methods employed during their education, or the contents of the pharmaceutical curriculum during their student years, which may not have sufficiently emphasized practical topic or even advances in inhaler technique and formulations over the years. For instance, Basheti et al. [38] observed a lack of practice in the correct use of inhalers in the current educational training standard in pharmaceutical studies. Moreover, most pharmacy students in their study indicated that the absence of inhalation practice and insufficient training were the main obstacles to demonstrating the correct inhalation technique. Therefore, hands-on inhaler training is necessary to improve inhalation technique demonstration skills [35, 37], which is also crucial for the postgraduate education of pharmacists. Numerous studies suggest that pharmacists’ participation in continuous education initiatives can positively impact their self-efficacy and professional behaviors [39, 40]. The results of Basheti et al. also confirmed a significant relationship between the knowledge of physicians and pharmacists about asthma and their inhaler demonstration skills [41]. In this context, it was noted that appropriate training, coupled with the provision of effective educational tools on asthma and COPD, could contribute to pharmacists playing a key role in patient education.

A crucial aspect of course evaluation is participants’ satisfaction [42]. Therefore, during our training, participants were given the opportunity to express their opinion on the course - whether it met their expectations and what they would be interested in for the future. The majority of pharmacists expressed high satisfaction with the training, concluding that it met their expectations and had practical significance. Providing appropriate training modules for pharmacists on patient education may, in turn, constitute a way to improve treatment effectiveness. If the pharmacy staff does not know how to use the inhaler properly, it may limit their ability to assist patients with asthma and COPD when necessary. The consequences of this issue can be significant, as asthma patients may misuse their inhalers from the time of prescription until they are seen again by a specialist physician. Also, due to the resulting lack of symptom control, physicians may prescribe increased doses of inhalers, leading to elevated costs and potential side effects [43]. Participation in additional training in pharmaceutical care and professional counseling is an excellent way to increase knowledge that is useful at the community pharmacy and reduce barriers related to perceived knowledge gaps [43–46]. In various studies, pharmacists were shown to believe that promoting specialist education for pharmacy staff will facilitate the counseling process [47, 48]. In the presented study, most participants indicated that additional practical workshops increase their knowledge, which may translate into better patient care at the pharmacy. This is in line with other studies [44, 47, 49–51]. Pharmacists’ education is beneficial for improving competencies, providing the ability for critical thinking, and enhancing problem-solving skills with decision-making during pharmacotherapy alongside physicians [48, 52].

Not only the pharmacist’s knowledge but also the way it is communicated to the patient has an impact on improving the effectiveness of therapy. Combining oral information with alternative, modern methods, such as an information leaflet or a practical demonstration of the equipment, can enhance the technique of using the inhaler. Pharmacists can play a key role in improving the outcomes of inhalation therapy by demonstrating inhalation techniques [53]. The use of demo devices contributes to improving the technique of using the pMDI in asthma patients [54]. The combination of written and oral instructions, along with a physical display by pharmacists, also helps reduce technical errors [55]. A similar result was observed in patients using DPI (type of DPI inhaler - turbuhaler) for asthma [24]. The combined oral explanation and demonstration by pharmacists may, therefore, be useful in improving the use of the inhaler, potentially leading to better therapeutic effectiveness [53].

## Conclusions

The postgraduate education course on inhaler use techniques for pharmacists, as presented in this study, received positive assessments from its participants. It not only met their educational expectations but was also effective in enhancing their knowledge and awareness of the subject. Considering the expressed interest and additional requirements from the participants, as well as the expanding scope of their professional responsibilities, it appears necessary to arrange similar initiatives in Poland to provide optimal support for pharmacists as they transition from a drug-centered approach to a more patient-centered one, aligning with the principles of pharmaceutical care. Meanwhile, as reports from other authors indicate, such a broader involvement of pharmacists in patient care may bring numerous benefits to both the healthcare sector and other professionals, saving their time and reducing workload. Better preparation of pharmacists in this aspect would result in more professional advice given to patients with asthma or COPD and, consequently, lead to better patient outcomes.

## Limitations

Participation in the survey was voluntary, therefore, some questions were omitted when the participants completed them. There was a definite predominance of women in the training. This is the same as the latest report of the Central Statistical Office in Poland for 2021. The vast majority of those working in pharmacies were women − 82.8% [56]. Participants had a knowledge test before and after the training, in which only their theoretical knowledge was checked. On the other hand, practical skills (e.g., handling inhalers, patient education) were supervised and corrected by the trainers during the workshops. In subsequent studies, it would be worth repeating the knowledge test after a longer period of time to assess knowledge retention and assess how often it is worth repeating the course. The pre-survey was conducted during the workshop; hence, the needs assessment was limited and can be refined for future projects.

.

## Data Availability

The data presented in this study are available on request from the corresponding author.

## Abbreviations

COPD: Chronic obstructive pulmonary disease
DPI: Dry powder inhaler
pMDI: Pressurized metered dose inhaler
SOP: Standard operating procedures

## References

[1] Lavorini F, Magnan A, Christophe Dubus J, Voshaar T, Corbetta L, Broeders M. i in. Effect of incorrect use of dry powder inhalers on management of patients with asthma and COPD. Respir Med. 2008;102(4):593–604. 10.1016/j.rmed.2007.11.003 18083019

[2] Axtell S, Haines S, Fairclough J (2017). Effectiveness of various methods of Teaching proper inhaler technique: the importance of Pharmacist Counseling. J Pharm Pract.

[3] Basheti IA, Salhi YB, Basheti MM, Hamadi SA, Al-Qerem W (2019). Role of the pharmacist in improving inhaler technique and asthma management in rural areas in Jordan. Clin Pharmacol Adv Appl.

[4] Dąbrowiecki P, Dąbrowski A, Gawlik R et al. What errors occur in asthma treatment in Poland? Lekarz POZ. 2021;7(2).

[5] Price D, Bosnic-Anticevich S, Briggs A (2013). Inhaler competence in asthma: common errors, barriers to use and recommended solutions. Respir Med.

[6] Padmanabhan M, Tamilarasu K, Rajaram M (2019). Inadequate inhaler technique, an everlasting problem, is associated with poor disease control– a cross sectional study. Adv Respir Med.

[7] Melani AS, Bonavia M, Cilenti V, Cinti C, Lodi M, Martucci P, Serra M, Scichilone N, Sestini P, Aliani M, Neri M, Gruppo Educazionale Associazione Italiana Pneumologi Ospedalieri (2011). Inhaler mishandling remains common in real life and is associated with reduced disease control. Respir Med.

[8] Sanchis J, Gich I, Pedersen S, Aerosol Drug Management Improvement Team (ADMIT) (2016). Systematic review of errors in Inhaler Use. Has Patient Technique Improved Over Time? Chest.

[9] Castel-Branco MM, Fontes A, Figueiredo IV (2017). Identification of inhaler technique errors with a routine procedure in Portuguese community pharmacy. J Pharm Pract.

[10] Al Rahbi HA, Al-Sabri RM, Chitme HR (2014). Interventions by pharmacists in out-patient pharmaceutical care. Saudi Pharm J.

[11] van der Molen T, van Boven JFM, Maguire T, Goyal P, Altman P (2017). Optimizing identification and management of COPD patients - reviewing the role of the community pharmacist. Br J Clin Pharmacol.

[12] Waszyk-Nowaczyk M, Bajsert A (2018). Profesjonalne Doradztwo w aptekach ogólnodostępnych - analiza wybranych krajów. Pol Prz Nauk Zdr.

[13] Jia X, Zhou S, Luo D, Zhao X, Zhou Y, Cui YM. Effect of pharmacist-led interventions on medication adherence and inhalation technique in adult patients with asthma or COPD: a systematic review and meta-analysis. J Clin Pharm Ther Published Online Febr. 2020;27. 10.1111/jcpt.13126. 10.1111/jcpt.13126 32107837

[14] Müller T, Müller A, Hübel C, Knipel V, Windisch W, Cornelissen CG, Dreher M (2017). Optimizing inhalation technique using web-based videos in obstructive lung diseases. Respir Med.

[15] Abdulsalim S, Unnikrishnan MK, Manu MK, Alrasheedy AA, Godman B, Morisky DE (2018). Structured pharmacist-led intervention programme to improve medication adherence in COPD patients: a randomized controlled study. Res Social Adm Pharm.

[16] Nguyen TS, Nguyen TLH, Van Pham TT, Hua S, Ngo QC, Li SC (2018). Pharmacists’ training to improve inhaler technique of patients with COPD in Vietnam. Int J COPD.

[17] Osman A, Ahmed Hassan IS, Ibrahim MIM (2012). Are Sudanese community pharmacists capable to prescribe and demonstrate asthma inhaler devices to patrons? A mystery patient study. Pharm Pract.

[18] Waszyk-Nowaczyk M, Guzenda W, Targosz P, Byliniak M, Plewka B, Dąbrowiecki P, Michalak M, Bąbol A, Szapel K, Bielas M, Żabiński J (2022). The influence of the pharmacists’ training on the quality and comprehensiveness of professional advice given in the field of inhalation techniques in Community pharmacies in Poland. Int J Environ Res Public Health.

[19] Ustawa z dnia 10 grudnia 2020 r. o zawodzie farmaceuty https://isap.sejm.gov.pl/isap.nsf/download.xsp/WDU20210000097/U/D20210097Lj.pdf (accessed on 12.08.2023)

[20] Regulation of the Minister of Science and Higher Education of July 26, 2019 on standards of education preparing to practice the profession of doctor, dentist, pharmacist, nurse, midwife, laboratory diagnostician, physiotherapist and paramedic https://isap.sejm.gov.pl/isap.nsf/download.xsp/WDU20190001573/O/D20191573.pdf

[21] Ilardo ML, Speciale A (2020). The Community Pharmacist: Perceived barriers and patient-centered Care Communication. Int J Environ Res Public Health.

[22] Kopciuch D, Paczkowska A, Zaprutko T, Ratajczak P, Nowakowska E, Kus K (2021). A survey of pharmacists’ knowledge, attitudes and barriers in pharmaceutical care concept in Poland. BMC Med Educ.

[23] Basheti IA, Reddel HK, Armour CL (2005). Counseling about turbuhaler technique: needs assessment and effective strategies for community pharmacists. Respir Care.

[24] Basheti IA, Armour CL, Reddel HK (2009). Long-term maintenance of pharmacists’ inhaler technique demonstration skills. Am J Pharm Educ.

[25] Bosnic-Anticevich SZ, Sinha H, So S (2010). Metered-dose inhaler technique: the effect of two educational interventions delivered in community pharmacy over time. J Asthma.

[26] Press VG, Arora VM, Shah LM (2012). Teaching the use of respiratory inhalers to hospitalized patients with asthma or COPD: a randomized trial. J Gen Intern Med.

[27] Waszyk-Nowaczyk M, Żabiński J, Plewka B (2019). Astma i POChP. Profesjonalne Wsparcie pacjenta w aptece. Obsługa inhalatorów - Podręczny poradnik Farmaceuty.

[28] Global Strategy for Asthma Management and Prevention, Report. 2020. https://ginasthma.org/wp-content/uploads/2020/04/GINA-2020-full-report_-final-_wms.pdf (accessed 03.05.22).

[29] Basheti IA, Armour CL, Bosnic-Anticevich SZ, Reddel HK (2008). Evaluation of a novel educational strategy, including inhaler-based reminder labels, to improve asthma inhaler technique. Patient Educ Counsel.

[30] Harden RM (1986). Ten questions to ask when planning a course or curriculum. Med Educ.

[31] McLean M, Gibbs T (2010). Twelve tips to designing and implementing a learner-centred curriculum: Prevention is better than cure. Med Teach.

[32] Mohamed Ibrahim OH (2012). Assessment of Egyptian pharmacists’ attitude, behaviors, and preferences related to continuing education. Int J Clin Pharm.

[33] Drug reviews - courses., not studies. Available online: https://aptekarski.com/artykul/przeglady-lekowe-kursy-nie-studia (accessed on 12.08.2023).

[34] Knowles MS. The Modern Practice of Adult Education. From Pedagogy to Andragogy; The Association Press: New York, NY, USA, 1980; ISBN 0-69-581472-9.

[35] Cerbin-Koczorowska M, Przymuszała P, Waszyk-Nowaczyk M, Plewka B, Marciniak R (2020). The need for simulated patient method implementation in Pharmaceutical Education in Poland. Indian J Pharm Educ.

[36] Poznań: a modern Medical Simulation Center will be built. Available online: https://www.prawo.pl/zdrowie/poznan-powstanie-nowoczesne-centrum-symulacji-medycznej,254201.html (accessed on 12.08.2023).

[37] Sufi S, Nenadic A, Silva R, Duckles B, Simera I, de Beyer JA, Struthers C, Nurmikko-Fuller T, Bellis L, Miah W, Wilde A, Emsley I, Philippe O, Balzano M, Coelho S, Ford H, Jones C, Higgins V. (2018). Ten simple rules for measuring the impact of workshops. PLoS Comput Biol. 14(8), e1006191. 10.1371/journal.pcbi.1006191 PMC6116923 30161124

[38] Basheti I, Natsheh A, Ammari W, Khater S, Qunaibi E, Bosnic-Anticevich S (2015). Education on correct inhaler technique in Pharmacy schools: barriers and needs. Trop J Pharm Res.

[39] Barnard M, White A, Bouldin A. Preparing pharmacists to care for patients exposed to intimate Partner violence. Volume 8. Pharmacy; 2020. p. 100. 10.3390/pharmacy8020100 PMC7357068 32531936

[40] Martin BA, Bruskiewitz RH, Chewning BA (2010). Effect of a tobacco cessation continuing professional education program on pharmacists’ confidence, skills, and practice-change behaviors. J Am Pharm Assoc.

[41] Basheti IA, Hamadi SA, Reddel HK (2016). Inter-professional education unveiling significant association between Asthma knowledge and inhaler technique. Pharm Pract (Granada).

[42] Cerbin-Koczorowska M, Przymuszała P, Fabianowska S, Gałązka N, Zielińska-Tomczak Ł (2022). Learning Theory-Driven Tips for Designing Effective Learning solutions for the Continuous Education of Community pharmacists to enhance patient-centered Care—A qualitative study. Healthcare.

[43] Bridgeman MB, Wilken LA (2021). Essential role of pharmacists in Asthma Care and Management. J Pharm Pract.

[44] Mohammed SI, Dawood EB, Abaas IS (2019). Perceptions and attitudes of community pharmacists’ towards patient counseling and continuing pharmacy education programs in Iraq. Iraqi J Pharm Sci.

[45] Palaian S, Rao PGM, Nair NM, Nair NM, Alam K (2008). Perception of community pharmacists towards patient counseling in South India. Pharma J Kenya.

[46] Sancar M, Okuyan B, Apikoglu-Rabus S, Izzettin FV (2013). Opinion and knowledge towards pharmaceutical care of the pharmacists participated in clinical pharmacy and pharmaceutical care continuing education program. Turk J Pharm Sci.

[47] Alshakka M, Bahattab A, Ravi Shankar P, Ansari M, Ali HS, Ibrahim MIM (2018). Perception of community pharmacy personnel towards patient counseling and continuing pharmacy education programs in Aden, Yemen. J Clin Diagn Res.

[48] Schindel TJ, Kehrer JP, Yuksel N, Hughes CA (2012). University-based continuing education for pharmacists. Am J Pharm Educ.

[49] Hamadouk RM, Yousef BA, Albashair ED, Mohammed FM, Arbab AH (2023). Perceptions of Community Pharmacists towards Patient Counseling and Continuing Pharmacy Education Programs in Sudan. Integr Pharm Res Pract.

[50] Poudel A, Khanal S, Kadir A, Palaian S (2009). Perception of Nepalese community pharmacists towards patient counseling and continuing pharmacy education program: a multicentric study. J Clin Diagn Res.

[51] Rajiah K, Preet Kaur K, Sivarasa S, Leowyeow M (2014). Perception of community pharmacists towards patient counseling and continuing pharmacy education program in Kuala Lumpur and Selangor states of Malaysia. Am J Pharm Heal Res.

[52] Kennerly J, Weber R (2013). Role of pharmacy education in growing the pharmacy practice model. 48. Hosp Pharm.

[53] Tamiya H, Mitani A, Abe T, Nagase Y, Suzuki H, Jo T, Tanaka G, Nagase T (2022). Association between Inhalation Instruction Method in Community pharmacies and inhaler device handling error in patients with obstructive lung disease: an evaluation of the impact of practical demonstration by pharmacists. Biol Pharm Bull.

[54] Self TH, Brooks JB, Lieberman P, Ryan MR (1983). The value of demonstration and role of the pharmacist in teaching the correct use of pressurized bronchodilators. Can Med Assoc J.

[55] Bosnic-Anticevich SZ, Sinha H, So S, Reddel HK (2010). Metered-dose inhaler technique: the effect of two educational interventions delivered in community pharmacy over time. J Asthma.

[56] Central SO, Poland. 2022. https://stat.gov.pl/obszary-tematyczne/zdrowie/zdrowie/zdrowie-i-ochrona-zdrowia-w-2021-roku,1,12.Html (accessed on 12.08.2023).

